# FASCE, the benefit of spironolactone for treating acne in women: study protocol for a randomized double-blind trial

**DOI:** 10.1186/s13063-020-04432-w

**Published:** 2020-06-25

**Authors:** Alexandra Poinas, Marie Lemoigne, Sarah Le Naour, Jean-Michel Nguyen, Solène Schirr-Bonnans, Valery-Pierre Riche, Florence Vrignaud, Laurent Machet, Jean-Paul Claudel, Marie-Thérèse Leccia, Ewa Hainaut, Nathalie Beneton, Cécile Dert, Aurélie Boisrobert, Laurent Flet, Anne Chiffoleau, Stéphane Corvec, Amir Khammari, Brigitte Dréno

**Affiliations:** 1 Clinical Investigation Centre CIC1413, CHU Nantes and INSERM, Nantes, France; 2 Dermatology Department, CHU Nantes, Nantes University, CRCINA, Nantes, France; 3 Department of Epidemiology and Biostatistics, CHU Nantes, Nantes, France; 4 Service Evaluation Economique et Développement des Produits de Santé, Département Partenariats et Innovation, Centre Hospitalier Universitaire de Nantes, Nantes University, Nantes, France; 5 Department of Dermatology, CHU Tours, INSERM U1253, University of Tours, Tours, France; 6 Private Practice, Tours, France; 7 Department of Dermatology, Allergology and Photobiology, CHU A. Michallon, Grenoble, France; 8 Service de Dermatologie, Poitiers University Hospital, Poitiers, France; 9 Service de Dermatologie, Le Mans Hospital, Le Mans, France; 10 Department of Pharmacy, CHU Nantes, Nantes, France; 11 Direction de la Recherche, Département Promotion, Cellule Vigilances, Centre Hospitalier Universitaire de Nantes, Nantes, France; 12 CHU Nantes, Service de Bactériologie-Hygiène Hospitalière, CRCINA, INSERM, U1232, Université de Nantes, Nantes, France

**Keywords:** Acne vulgaris, Spironolactone, Cycline

## Abstract

**Background:**

Acne vulgaris has increased in women over the past 10 years; it currently affects 20–30% of women. The physiopathology of adult female acne is distinguished from that of teenagers essentially by two factors: hormonal and inflammatory. On a therapeutic plan, the four types of systemic treatment approved for female acne include cyclines (leading to bacterial resistance); zinc salts (less effective than cyclines); and antiandrogens (risks of phlebitis). The last alternative is represented by isotretinoin, but its use in women of childbearing potential is discouraged because of the teratogen risks. In this context, spironolactone could represent an interesting alternative. It blocks the 5-alpha-reductase receptors at the sebaceous gland and inhibits luteinizing hormone (LH) production at the pituitary level. It has no isotretinoin constraints and does not lead to bacterial resistance. Currently, very few studies have been performed in a limited number of patients: the studies showed that at low doses (lower than 200 mg/day), spironolactone can be effective against acne. In that context, it is clearly of interest to perform the first double-blind randomized study of spironolactone versus cyclines, which remains the moderate acne reference treatment, and to demonstrate the superiority of spironolactone’s efficacy in order to establish it as an alternative to cyclines.

**Methods:**

Two hundred female patients will be included. They must have acne vulgaris with at least 10 inflammatory lesions and no more than 3 nodules. After randomization, the patients will be treated by spironolactone or doxycycline for 3 months and after placebo. The study will be blind for the first 6 months and open for the last 6 months.

**Discussion:**

The treatment frequently used in female acne is systemic antibiotics with many courses, as it is a chronic inflammatory disease. In the context of the recent World Health Organisation (WHO) revelation about the serious, worldwide threat to public health of antibiotic resistance, this trial could give the physician another alternative in the treatment of adult female acne instead of using isotretinoin, which is more complex to manage.

**Trial registration:**

ClinicalTrials.gov: NCT03334682. Registered on 7 November 2017.

## Administrative information

Note: the numbers in curly brackets in this protocol refer to SPIRIT checklist item numbers. The order of the items has been modified to group similar items (see http://www.equator-network.org/reporting-guidelines/spirit-2013-statement-defining-standard-protocol-items-for-clinical-trials/).

**Table:** 

**Title {1}**	Randomized double-blind study on the benefit of spironolactone for treating acne in adult females
**Trial registration {2a and 2b}**	Registration number NCT03334682, first published on 7 November, 2017 https://clinicaltrials.gov/ct2/show/NCT03334682?term=Dreno&cond=acne&draw=2&rank=2
**Protocol version {3}**	The updated protocol is at version 8 on 26 November, 2019.
**Funding {4}**	This study is supported by a grant from the French Ministry of Health awarded in 2016 (under the Hospital Clinical Research Programme), number 16–0290. This grant has funded the FASCE clinical trial, for which Prof. Dreno is the coordinating investigator. This grant was allocated following peer review. The research projects selected by this call for tenders must contribute to medical progress and the improvement of the healthcare system. The experts’ comments have been taken into account in the final protocol submitted to the regulatory authorities. The funding body will be mentioned in the acknowledgements as having funded the research but it is not involved in the study, analysis or interpretation of the data.
**Author details {5a}**	AP wrote the manuscript. AK, BD, SSB, VPR, FV, AC and JMN assisted with the drafting of the manuscript. AP, BD, AK, BD, SSB, VPR, FV, AC, LF, ML, SLN, SC and JMN designed the trial. BD, AP, JMN, VPR, AC and LF wrote the protocol and/or the file for the experimental drug and assisted with the drafting of the manuscript. CD coordinated the submission of the protocol and the follow up of (1) the Health Ministry’s tender and (2) the regulatory authorities, and coordinated the trial. JMN wrote the methodological/statistical analyses in the protocol. VPR and SSB wrote the health economic analyses. ML, SLN, LM, JPC, MTL, EH, NB, AB and BD participated in patient enrolment and follow up. AC assisted with pharmacovigilance for the trial.
**Name and contact information for the trial Sponsor {5b}**	Cecile Dert is the Sponsor project manager and she is coordinating the logistics of the trial.
**Role of Sponsor {5c}**	All the submissions/declarations were made by the Sponsor Department at CHU Nantes, which of course manages the quality of the data collected. The data collected during the study will be processed electronically in accordance with the requirements of CNIL, the French Data Protection Authority, and with the European and French regulations regarding safety concerns. Requests for substantial modifications of the protocol should be addressed to the Sponsor for approval or notification to the French regulatory authorities and/or the Ethical Review Board concerned in compliance with Law 2004–806 of 9 August, 2004 and its implementing decrees. As already mentioned, this project was awarded a grant from the Ministry of Health in 2016. Funding is in tranches according to the progress of the project. Note that the funding body has no relation to the data collected or to the protocol and its amendments.
**Financial and other competing interests for principal investigators for the overall trial and each study site {28}**	No financial or competing interests for the investigators have to be declared.

## Introduction

### Background and rationale{6a}

To understand exactly what acne vulgaris is, we must begin below the skin’s surface, deep in the hair follicle. The follicle, which is lined with skin cells, contains sebaceous glands that produce oil (sebum). Normally the skin cells that line the follicle are shed and brought to the skin’s surface by the sebum and are then washed away. However, when the cells stick together instead of shedding, they form a plug or blockage. A clogged pore is a commonly used term for a plugged follicle. Beneath the plug, a sac is formed (known as a microcomedone) that contains dead skin cells and oil. Bacteria (*Cutibacterium acnes*) grow freely in this environment, feeding on the dead skin cells and oil. As the sac continues to grow, either a whitehead (known as a closed comedone), or a blackhead (open comedone) forms. In more serious cases, the sac will become larger, encouraged by the cells that the body sends into the sac to fight the infection. Inflammation will result, and a bump (papule or pustule), painful nodule or cyst will develop.

Though acne becomes less common in adulthood than in adolescence, nearly half of people in their twenties and thirties continue to have it. About 4% continue to have difficulties into their forties [1].

Acne vulgaris has increased in women over the past 10 years. The prevalence is currently 15–50% across different studies [2–4]. Acne in women is now a well-known entity considered as different to adolescent acne [5, 6]. It is reported to most often affect women aged 25 years or older. A recent study isolated two distinct types of clinical presentation: (1) the first is similar to teenager acne, with diffuse acne lesions over the face including hyperseborrhea, retentional lesions (mainly closed comedone) and superficial inflammatory lesions and (2) the second is always mild-to-moderate acne located on the inferior third of the face (mandibular location), mainly with few deep, inflammatory chronic cysts [7].

The physiopathology of adult female acne is characterized mainly by two specific factors:

1. The hormonal factor suggested by the premenstrual flare-up of acne lesions in more than 60% of women [2, 8] and by the efficacy of anti-androgens and fourth-generation contraception (non-androgenic progestin). The androgen level in blood, previously investigated in women with acne, shows that women with abnormalities are between 7.6% [7] and 86% [9, 10], according to the studies. This strong difference can be explained by the different profiles of female acne included in the trials. Some studies included women with endocrine illnesses associated with hirsutism, irregular cycles and acne. In one study, the serum sex hormone binding globulin (SHBG) values correlated negatively with acne severity, possibly due to an increased level of free testosterone [11]. On the other hand, three other studies did not show any link between androgen level and acne severity [9, 12, 13]. When women with acne with a control population, higher levels of dehydroepiandrosterone (DHEAS) [10, 12], delta-4 androstendione [10], dihydrotestosterone (DHT) [13] and free testosterone [10, 13] have been noted. Last, DHEAS, DHT and insulin-like growth factor-1 (IGF-1) serum levels correlate positively with acne lesion counts in female acne [14]. However, in the majority of women in whom acne is the sole clinical sign (no other clinical sign of hyperandrogenia), no abnormal hormone profiles are present. Two of the main hypotheses discussed today are hypersensitivity of androgen receptors identified in sebocytes and keratinocytes or hyperactivity of enzyme metabolizing androgens in these cells: the term peripheral hyperandrogenia is proposed.
2. Chronic activation of innate immunity of the skin linked to the presence of resistant *C. acnes* strains in the pilosebaceous follicle. In the great majority of women who have had acne for many years, frequent prescriptions of topical antibiotics (erythromycin, clindamycin) induce the development of *C. acnes* and *Staphylococcus* resistant strains.

Four types of systemic treatment are approved in acne [15]:

1. Cyclines, which can induce modifications of the cutaneous microbiome and bacterial resistance with a risk of non-clinical response [16].
2. Zinc salts, which target only the inflammatory lesions and were shown to be less effective than cyclines [17].
3. Antiandrogens, such as acetate of cyproterone, which are associated with risks of phlebitis, pulmonary embolism, hypertriglyceridemia and hypercholesterolemia.
4. Isotretinoin, which is a teratogenic drug requiring strict use of contraception, with monthly pregnancy testing and testing of cholesterol, triglycerides and liver enzymes. It can also be associated with depression in some patients. In addition, even if the risk of relapse is low after a course of isotretinoin (less than 20%), it has been shown to be higher in women with hyperandrogenia [18]. Thus, even if isotretinoin, by inducing apoptosis of the sebaceous glands, is the most effective drug in acne, its use is sometimes questionable.

Note that the direct cost of acne in the USA is estimated to exceed USD 1 billion per year, with USD 100 million spent on over-the-counter products [19]. Despite this high cost, 81% of women report failures with systemic antibiotics and failures in response to treatment with isotretinoin ranging from 15 to 30% [20].

In this context, spironolactone may represent an interesting alternative. It blocks the 5-alpha-reductase receptors at the sebaceous gland and inhibits luteinizing hormone (LH) production at the pituitary level [21]. It is not subject to the constraints of isotretinoin, does not lead to bacterial resistance and targets peripheral hyperandrogenism.

Currently, 10 randomized controlled trials (RCT) have been performed in a small number of women with acne [22]. Between 10 and 66 female patients with acne were treated by spironolactone. These 10 RCTs included 16 comparisons of spironolactone with placebo or active treatment and all these trials were considered to have a high risk of bias (*inter alia* selection, detection and/or reporting bias). The interest is in the fact that low doses of spironolactone at 50–200 mg per day (with one study using spironolactone at the dose of 100 mg per day but for 16 days per month [23]) appear to induce clinical response in acne. The endpoint varied between studies but was most frequently the lesion count [24–26]. In all the studies published, spironolactone was effective in treating acne lesions located on the face [27] and/or on the back [28–30]. In the largest study published until now, Shaw et al. treated 85 women with spironolactone at 50–100 mg/day either as single-drug therapy or as an adjunctive treatment in an open study. They reported a complete response in treating acne lesions in one third of the cases, a marked improvement in another third, and a partial improvement in the final third of patients [8]. Notably, all these studies were performed in an open setting with no control group. The tolerance of the treatment was excellent. The most frequent adverse events were fatigue and menstrual irregularities, but they were always grade 1 with no need of treatment interruption.

In that context it appears of particular interest to perform the first double-blind randomized study comparing spironolactone with cycline as:

- Some open trials indicate that spironolactone is effective in treating female acne, and could represent an interesting alternative to systemic treatments such as antibiotics or isotretinoin.
- No randomized trials comparing spironolactone with cycline, which remains the reference treatment for moderate acne, have been performed
- There is no labelling for acne for spironolactone.

The objective is to demonstrate the superiority of spironolactone’s efficacy in treating adult female acne in order to establish it as an alternative to cycline that could be prescribed by the dermatologist with the advantage of being inexpensive.

### Originality and innovative aspects of the FASCE trial

In spite of numerous scientific references to the efficacy of spironolactone in adult female acne, no RCT has ever been performed to demonstrate its efficacy in comparison with the treatment usually used. Furthermore, the acne treatment frequently used in this indication is an antibiotic treatment, and World Health Organisation (WHO) recently revealed the serious, worldwide threat to public health of antibiotic resistance (WHO First Global on antibiotic resistance - 30 April 2014 [31]).

It is important to find an effective and cheap treatment without using antibiotics. Acne is not lethal but it affects at least 15% of the adult female population and it causes serious problems in daily life with societal impact. This trial could give the dermatologist another alternative to systemic antibiotics before prescribing isotretinoin, for which the management is more complex.

Another original aspect of this study is the use of specific evaluation scales that are more accurate for use in adult female acne. In the past, several grading systems have been described [32]: more than 25 methods of assessing acne severity and more than 19 methods for counting lesions are available. In 1997, for example, Doshi, Zaheer and Stiller devised a global acne grading system (GAGS) [33]. Using this system the face, chest and back are divided into six areas (forehead, each cheek, nose, chin, chest and back) and a factor of 1, 2 or 3 is assigned to each area, based on size. Currently, the commonly used scales for assessing acne in France and in Europe are the Global Evaluation Assessment (GEA) [34] or the *Echelle de Cotation des Lésions d’Acné* (ECLA) [35] as proposed by our team. However, they were designed for treatment of acne in adolescents, and are focused on the lesions located on the face.

As detailed above, women frequently have lesions in the mandibular region, meaning that the use of standard assessment scales could underestimate their lesions. Recently, our new scoring tool, the Adult Female Acne Scoring Tool (AFAST) has been proposed for this purpose [36]. The AFAST scale is composed of the GEA scale to assess acne on the face and a second scale developed to assess acne in the mandibular zone [7]. Thus, the use of currently available scales considering the whole face to assess the severity of acne underestimate the severity of this type of acne, scoring it most of the time as mild and sometimes as moderate. In addition, AFAST modulates results from acne grading on the full face (GEA - score 1) with that of the mandibular and submandibular zone (score 2), which conditions the choice of the most suitable treatment approach:

1. Score 1: the GEA, as stated, is a validated acne assessment scale [34]. It is based on the global assessment described by Thiboutot et al. [37].
2. Score 2: this score exclusively assesses acne in an area from the left and right mandibular zone to the upper edge of the trunk [36].

The use of this scoring tool will allow us to precisely evaluate the effect of spironolactone in the context of adult female acne.

To conclude, spironolactone has no official labelling for acne, making it difficult for the dermatologist to prescribe it in clinical practice and thus this randomized trial could have an impact on daily practice.

This clinical randomized trial will be stratified by the type of contraception used by the participants. As stated, another option in the arsenal of hormonal treatments is the use of oral contraceptives (OCs), which act largely by suppressing ovarian androgen production. Pills combining oestrogen and progestin are used for acne, as progestin-only and contraceptive implants may exacerbate the condition [38].

Earlier drugs (first and second generation) contain progestogen, derived from the so-called androgenic testosterone (levonorgestrel or norethisterone), with effects close to those of the male hormone and which can therefore aggravate the acne. The progestogen prevails over the estrogen and this pill has a progestational climate and favours acne in women who are subject to it. On the other hand, with third-generation progestogens (gestodene, norgestrel or desogestrel), i.e. those that are not androgenic, the oestrogen asserts itself and the drug climate is oestrogenic, which is desirable in cases of acne, but the effect is more often suspensive, with a relapse after stopping treatment. For fourth-generation drugs, an anti-androgenic progestogen (not just non-androgenic) has been chosen, which neutralizes the androgens. Nevertheless, because of the risk of thrombosis, the French Regulatory Authority (ANSM) advised physicians in December 2012 to systematically favour the second-generation contraceptive pill.

The female patients included in our trial will be women of childbearing potential, except for women who have undergone sterilization surgery or early menopause. They will be included in the study if they use a reliable method of contraception; those who use mechanical contraception or abstinence will not be included.

The women will be stratified on the basis of four strata following the acne effect foreseen by the contraception (some increase acne and others decrease it).:

1. 1st arm: implant, generation I and II OCs, progesterone intrauterine device or other kind of hormonal contraception (vaginal ring, patch, injection).
2. 2nd arm: copper intrauterine device (IUD) (hormone-free contraception).
3. 3rd arm: generation III and IV OCs.
4. 4th arm: no contraception (sterilization surgery or menopause).

### Objectives {7}

The primary objective was to demonstrate the superiority of spironolactone’s efficacy over doxycycline (reference treatment) in adult female acne.

The secondary objective is comparison of the following endpoints in the two study arms:

- Safety (clinical and biological) up to 12 months of follow up
- The percentage of patients with AFAST score 1 (GEA) at score 0 or 1 at 2, 4, 6, 9 and 12 months
- The percentage of patients with AFAST score 2 (mandibular) at score 0 or 1 at 2, 4, 6, 9 and 12 months
- Complete remission at 2, 4, 6, 9 and 12 months
- Quality of life at 2, 4, 6, 9 and 12 months

and further:

- Comparison of *C. acnes, Malassezia and S. epidermidis, aureus* at baseline (D0) and 4 months in each arm and between the two arms
- Comparison of inflammatory lesions of the face at D0 and 2, 4, 6, 9 and 12 months in each arm and between the two arms
- Comparison of retentional lesions of the face at D0 and 2, 4, 6, 9 and 12 months in each arm and between the two arms
- Comparison of the number of face lesions between D0 and 2, 4, 6, 9 and 12 months in each arm and between the two arms
- Comparison of trunk lesions (factor F2 of ECLA) between D0 and 2, 4, 6, 9 and 12 months in each arm and between the two arms
- Comparison of the percentage of patient relapses between the two arms at month 4 (M4) and month 6 (M6)
- Comparison of the percentage of patients in the two arms who have reappearance of 10% or more inflammatory lesions at M6
- Cost-effectiveness analysis (economic efficiency analysis) of spironolactone versus doxycycline at 6 months

### Trial design {8}

Our study is a randomized double-blind trial for the first 6 months (timeline for primary objective), and is an open trial for the last 6 months of follow up. The female patients will be randomized either to the acne routine-care arm, i.e. cycline (doxycycline 100 mg/day for 3 months followed by a placebo for 3 months) or to the experimental drug arm (spironolactone 150 mg/day for 12 months).

Blinding will be removed for all patients after 6 months of evaluation - once the primary endpoint has been measured - whatever the result, to limit the constraints for the patients in the doxycycline arm. If blinding were maintained, patients in the doxycycline arm would have to take a placebo for up to 6 months in addition to the 3 months already taken. The objective of the last 6 months of follow up is to assess the maintenance of each treatment’s efficacy during this time.

## Methods: participants, interventions and outcomes

### Study setting {9}

Patients will be recruited by the dermatology departments of several French reference centres, including study centres in western France belonging to the Dermatological Institute of the Western Region (IDGO), supported by GIRCI GO. The investigation centres are the dermatological departments of the university hospitals of Nantes, Brest, Le Mans, Grenoble and Tours, the regional hospital of La Rochelle and a private dermatologist in Tours.

### Eligibility criteria {10}

It is commonly acknowledged that acne affecting female patients under 20 years of age is considered to be “adolescent female acne”. Since this protocol concerns adult female acne, the recruited patients will be women aged at least 20 years. These patients will have acne vulgaris with at least 10 inflammatory lesions and no more than 3 nodules. The eligibility criteria are shown in Table 1.

**Table 1:** Inclusion and non-inclusion criteria of the FASCE trial

Inclusion criteria	Non-inclusion criteria
• Female patient ≥ 20 years old	• Patient affected by active/progressive diseases, as infections including hidradenitis suppurativa, cancers or endocrine syndrome (e.g. polycystic ovary syndrome)*,* Addison’s disease)
• Patient with acne, with at least 10 inflammatory lesions and no more than 3 nodules (GEA score between 2 and 4)	• Patient affected by rosacea (folliculitis)
• Patient who already had one cycline course for her acne treatment with a 3-month* wash out or who never took any cyclines	• Patient with contra-indication to the use of one of the investigational products or auxiliary: ◦ Patient with intolerance or hypersensitivity to cyclines, spironolactone or to any ingredient present in associated benzoyl peroxide gel ◦ Patient with significant impairment of renal excretory function, acute or chronic renal failure, anuria ◦ Patient with life-threatening or very severe hepatic impairment. (grade III or IV)
• Patient having signed an informed consent	• Patient with hyperkalaemia or strongly requiring potassium-sparing diuretics (e.g. amiloride, canrenoate, eplerenone, triamterene) or treated continuously with ACE inhibitors, angiotensin II antagonist, NSAIDs, heparin and molecular-weight heparin, ciclosporin and tacrolimus, or treated with lithium
• Absence of use of oral antibiotics and zinc salts in the last 30 days	• Patient requiring topical isotretinoin or who stopped this drug less than 2 weeks ago
• Absence of use of topical antibiotics in the last 15 days	• Association with potassium salts except in the case of hypokalaemia
• Absence of use of systemic isotretinoin and antiandrogens in the last 6 months (but antiandrogens used as contraceptive are authorized)	• Patient previously treated with spironolactone
• Absence of microphysiotherapy in the last 15 days	• Pregnant woman or likely to become pregnant or nursing and refusing to use an effective contraceptive method
• Women of child-bearing age under contraception for 3 months (oral contraception, implant, IUD or other kind of hormonal contraception)	• Patient participating in another interventional clinical trial
• Patients with social security	• Patient under guardianship or trusteeship

*GEA* Global Evaluation Assessment, *ACE* angiotensin-converting enzyme, *NSAID* non-steroidal anti-inflammatory drug, *IUD* intra-uterine device

### Who will take informed consent {26a}

Before proceeding with any examination related to the research, the investigator will obtain the patient’s freely given FASCE informed consent, in writing.

### Model consent form and other related documentation given to participants and authorized surrogates {32}

The Supplementary Material file contains the French informed consent form that the patient signs prior to their inclusion in the trial (version 2, updated 1 July 2017).

### Additional consent provisions for collection and use of participant data and biological specimens {26b}

Biological samples for bacterial strain identification will be collected and stored if the patient has signed the biocollection informed consent form. In this informed consent form it is noted that the samples may be used for other pieces of scientific research. This biocollection and consent procedure has been registered under number DC-2011-1399 by the French Ethics Committee: CPP Ouest IV.

## Interventions

### Explanation for the choice of comparators {6b}

As already stated, although acne is not an infectious disease, oral antibiotics have remained a mainstay of treatment over the last 40 years. The anti-inflammatory properties of oral antibiotics, particularly cyclines, are effective in treating inflammatory acne [39, 40].

All the guidelines - French [41], European [42] and American [43] - recommend cycline for first-line treatment but limited to a treatment period of 3 months to minimize the development of bacterial resistance. This is why the treatment duration of the comparator arm we chose is doxycycline 100 mg/day for 3 months. The guidelines specify also that monotherapy with a systemic antibiotic is not recommended. Concomitant topical therapy with, for example, benzoyl peroxide should be used with systemic antibiotics and for maintenance after completion of systemic antibiotic therapy. Patients in the two study arms will receive this topical therapy.

The primary objective is the efficacy of doxycycline or spironolactone seen at M4 and/or M6. To avoid results bias and because our experience shows that maximum efficacy is seen for cycline between M4 and M6, the best clinical response between M4 and M6 will be chosen for the primary endpoint.

The response of acne to spironolactone may not be optimal after only 3 months of treatment [22]. A retrospective trial reported by Burcke and Cunliffe showed an average reduction in acne that was optimal at month 6 [24].

**Fig. 1:**
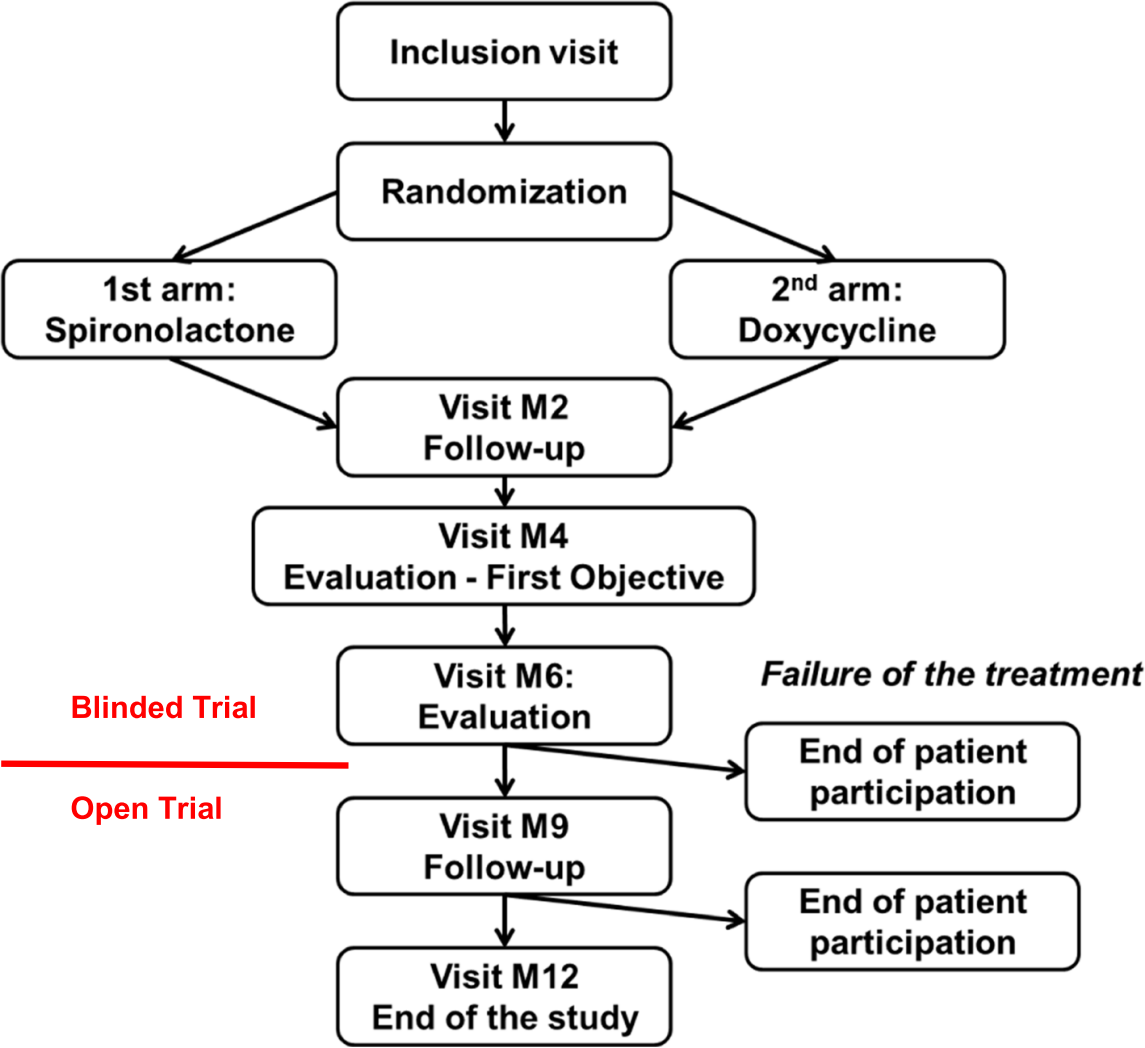
Study diagram

In conclusion, in order not to bias the study in either direction, regarding the primary objective, the efficacy of the two treatments will be measured at 4 and 6 months in each arm and the best results in the two arms will be compared (Fig. 1). At the end of the M6 visit, after the clinical evaluation, all the data will be accessible and open to the patient and the investigator. Patients with complete or partial remission will continue the trial. Other patients will drop out of the trial and be treated by the investigators according to their usual practice. The same approach will be used at the M9 visit: relapse patients will drop out of the trial and will be treated by the investigators according to their usual practice.

### Intervention description {11a}

As this is a double-blind study, the intervention drug, its comparator and the placebo will be managed by the pharmacist of the coordinating centre, CHU Nantes. The oral dose of 150 mg of spironolactone, once a day during the complete trial period has been chosen based on the literature and our own experience. Pills will be dispensed at each protocol visit (i.e. every 2 months for 12 months). The efficacy of spironolactone has been established by several studies that showed improvement, with reductions in lesions ranging from 50 to 100% in women treated for acne [44]. In most of these studies, the dosage range used was 100–200 mg daily, with response noted over approximately 3 months [44].

For the comparator, as recommended by the French Guidelines for acne treatment [41], the doxycycline treatment for acne vulgaris will be 100 mg administered once a day for 3 months. From M4 to M6, a placebo will be administered. Pills will be dispensed at each protocol visit (i.e. every 2 months for 6 months). The two treatments will be taken orally. As the antibiotics must be taken during a meal, and to retain the double-blind effect of our trial, the patients in the two arms should take their treatment during a meal. The treatment should be taken with a full glass of water and the patient should remain in the upright position for at least 1 h.

Monotherapy with a systemic antibiotic is not recommended [43]. Concomitant topical therapy with, for example, benzoyl peroxide 5%, should be used with systemic antibiotics and for maintenance after completion of systemic antibiotic therapy. Patients in the two arms will receive this topical therapy.

**Fig. 2:**
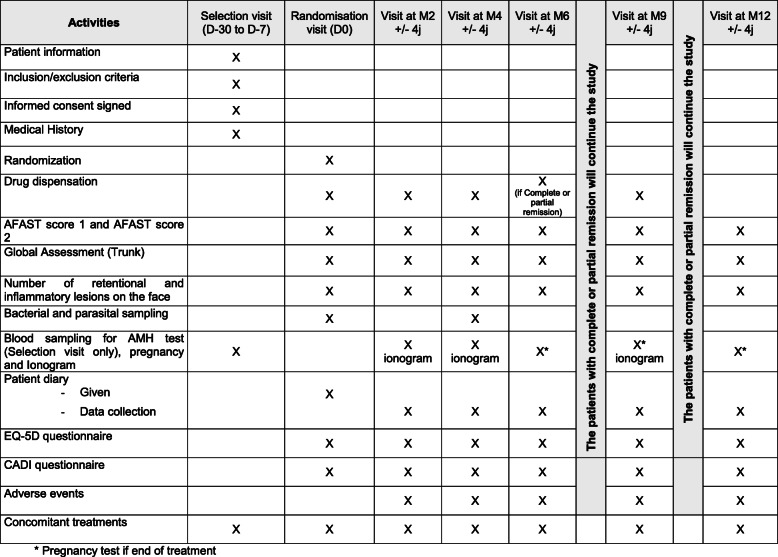
Study schedule. AFAST, Adult Female Acne Scoring Tool; EQ-5D, Euroqol-5 dimensions; CADI, Cardiff Acne Disability Index; D, day; M, month

The treatment continues after M6, which is the main objective to meet the secondary objectives. However, from the M6 visit, the treatment will be stopped in the event of failure to respond, the patient will be withdrawn from the study and the investigator will prescribe another treatment according to usual practice. In the case of treatment success, the patient in the spironolactone arm will continue with this treatment “open”, and the patient in the doxycycline arm will continue to participate only with the topical therapy*.* The schedule for the complete trial is shown in Fig. 2.

### Criteria for discontinuing or modifying allocated interventions {11b}

No dosage adjustment is required by the protocol. However, in case of hyperkalaemia, the spironolactone treatment will be adjusted. The patient must stop using the product and seek emergency medical attention immediately if she develops signs and symptoms of a serious hypersensitivity reaction such as throat tightness, difficulty breathing, feeling faint or swelling of the eyes, face, lips or tongue. Also, she must stop using the product if she develops hives or itching of the face or body.

### Strategies to improve adherence to interventions {11c}

Compliance will be assessed in the FASCE trial by return tablet count. At the M2, M4, M6, M9 and M12 visits, the patient will return the empty study drug packages and the investigator or a member of their team will count them.

### Relevant concomitant care permitted or prohibited during the trial {11d}

Due to spironolactone, anything that can induce hyperkalaemia is forbidden: potassium-sparing diuretics, potassium supplements and drugs such as ACE inhibitors, angiotensin II antagonists, non-steroidal anti-inflammatory drugs (NSAIDs), heparin and low molecular weight heparin, ciclosporin, tacrolimus and trimethoprim. Lithium is also not recommended in this trial. Concurrent use of lithium and spironolactone may result in increased lithium concentrations and lithium toxicity due to decreased lithium excretion. So, the monitoring of lithium plasmatic concentration will be reinforced.

### Provisions for post-trial care {30}

At the end of the clinical research, the patient will be followed by her dermatologist and will benefit from the usual care of her disease. The Sponsor will take out an insurance policy covering the financial consequences of its civil liability in compliance with the regulations.

### Outcomes {12}

The primary endpoint corresponding to the treatment efficacy is determined by the probability of success in each arm. In order to reduce the variability related to the fluctuation over time of the therapeutic response, we took the maximum value of this response. This maximum value appears between the fourth and sixth month of treatment. We could also have taken the average measured over 2 months, but clinically this is not interesting because the optimal time of efficacy is different between the two treatments This probability of success (or relapse) is defined by a decrease in both AFAST scores: (1) AFAST score 1 (corresponding to the GEA score): decrease of at least two grades compared to baseline or to grade 0 if the baseline was at 1, and (2) AFAST score 2 (mandibular score): decrease to grade 1 if baseline was > 1 or to grade 0 if the baseline was at 1.

AFAST score 1 (also called GEA) assesses the comedones (open and closed), the non-inflammatory lesions, the papules and pustules and the nodules. The stage is defined according to a global evaluation of severity of acne and ranges from grade 0 (no acne) to grade 5 (the worst situation). AFAST score 2 assesses acne on an area from the left and right mandibular zone to the upper edge of the trunk and ranges from grade 0 (no acne) to grade 3 (the worst situation).

For the secondary endpoints:

1. Safety/tolerance: reporting of the number and type of adverse events (AEs) and serious adverse events (SAEs) up to month 12 of follow up and abnormal values of ionogram (sodium, potassium, chlorine and calcium) at 0, 2, 4 and 9 months. First the percentage of AEs and SAEs will be calculated then they will be classified according to the organ affected.
2. Efficacy: these outcomes correspond to the reporting of the:
  - Number of patients with AFAST score 1 (GEA) at 0 or 1, at M2, M4, M6, M9 and M12
  - Number of patients with AFAST score 2 (mandibular) at 0 or 1, at M2, M4, M6, M9 and M12
  - Number of patients with both AFAST scores 1 and 2 at 0 or 1, at M2, M4, M6, M9 and M12
  - Number of inflammatory lesions of the face at D0 (baseline), M2, M4, M6, M9 and M12
  - Number of retentional lesions of the face at D0, M2, M4, M6, M9 and M12
  - Number of face lesions at D0, M2, M4, M6, M9 and M12
  - Number of trunk lesions (factor F2 of ECLA) at D0, M2, M4, M6, M9 and M12
  - Number of patients with relapses at M4 and M6

The definition of relapse is the same as the primary endpoint:

1. AFAST score 1 (GEA score): increase of 2 points in the score at the previous visit, in the case of success, or
2. AFAST score 2 (mandibular score): increase of 1 point in the score at the previous visit, in the case of success
  Number of patients with reappearance of 10% or more of inflammatory lesions at M6

For these nine outcomes, percentages will be calculated at each time point:

- Quality of life (QoL): reporting of the scores of two QoL questionnaires: the Euroqol-5 dimensions (EQ-5D) and the Cardiff Acne Disability Index (CADI) at M2, M4, M6, M9 and M12. This outcome will be measured by the percentage of patients whose quality of life has improved relative to baseline.
- Microflora: bacterial and parasitic sampling at D0 and M4. The presence *of P. acnes, M. Furfur* and *S. epidermidis/ aureus* will be sought and a change in these populations from baseline will be measured at M4. A percentage of decrease will be calculated.
- Medico-economy: incremental cost-effectiveness ratio (cost per quality-adjusted life year (QALY)) of the comparison between spironolactone and cycline at 6 months. Criteria for the calculation of the cost-effectiveness ratio are detailed in “Methods for additional analyses”.

### Participant timeline {13}

The maximum duration of treatment per patient is 12 months, and the recruitment period is 41 months.

### Sample size {14}

For sample size estimation purposes, the targeted difference in the percentage of success is 70% in the experimental arm compared with 50% success in the control arm, indicating a relative increase in success equal to 40%. Assuming a type I error rate of 5% and at least 80% statistical power, the sample size required is at least 91 subjects per group. To take into account the possibility of patients being lost to follow up, 10% (9 subjects) will be added to each group. Finally, approximately 200 subjects will be randomized to the two arms in a 1:1 ratio.

### Recruitment {15}

Acne vulgaris is a common condition. However, it is more common in the private sector than in public hospitals. Some centres such as Nantes have a one-day consultation devoted to acne. A feasibility study performed in each centre showed that between 8 and 15 patients per centre could be included per year, making these recruitment targets achievable.

## Assignment of interventions: allocation

### Sequence generation {16a} and concealment mechanisms {16b}

This balanced block randomization is computer-generated. Subjects are randomized into blocks as the allocation progresses, a block being a subgroup of predetermined size within which there is a random allocation of patients. The software SAS version 9.4 is used for randomization. The randomization will be centralized and stratified based on the following four groups of based on the type of contraception used by the participants:

1. Strata 1: implant, generation I and II OCs, progesterone intrauterine device or other kind of hormonal contraception (vaginal ring, patch, injection)
2. Strata 2: copper intrauterine device (IUD) (hormone-free contraception)
3. Strata 3: generation III and IV OCs
4. Strata 4: no contraception (sterilization surgery or menopause)

### Implementation {16c}

The randomization key is known only to the biostatistician and data managers, to make it impossible for the investigator to assign a specific treatment. At the randomization visit (day 0) the investigator will check the inclusion and non-inclusion criteria, and then validate the randomization on the electronic case report form (eCRF). Drug prescriptions (blindspironolactone/doxycycline) will be made out for 2 months. In addition, a concomitant topical therapy, benzoyl peroxide 5%, will be given.

## Assignment of interventions in a double-blind method

### Who will be blinded {17a}

Our study is a randomized double-blind trial during the first 6 months (timeline for the primary objective), and an open tria during the last 6 months of follow up. The female patients will be randomized either to the acne routine care arm, i.e. cycline (doxycycline 100 mg/day for 3 months followed by a placebo for 3 months) or to the experimental drug arm (spironolactone 150 mg/day for 12 months).

The patients and the investigators and their teams will only be blinded to the treatment assigned during the first 6 months. As Karanicolas et al. pointed out in their article from 2010, their study should have been masked to the biostatisticians until analyses had been performed, to reduce the study bias [45].

Blinding will be removed for all patients after 6 months of evaluation - once the primary endpoint has been measured - whatever the result, to limit the constraints for the patients in the doxycycline arm. If blinding were maintained, patients in the doxycycline arm would have to take a placebo for up to 6 months in addition to the 3 months already taken. The objective of the last 6 months of follow up is to assess the maintenance of the efficacy of each treatment during this time.

### Procedure for unblinding if needed {17b}

The investigator should inform the Sponsor if unblinding is deemed to be necessary due to an emergency. Unblinding will be performed via the eCRF thanks to a detailed procedure that will be given to each site. Each decoding will be tracked and classified in the investigator’s management file and in the trial management file.

## Data collection and management

### Plans for the assessment and collection of outcomes - description of the parameters for evaluating efficacy {18a}

The first endpoint is the new validated score for adult female acne: the Adult Female Acne Scoring Tool (AFAST) [36]. The AFAST uses two independent scores: score 1 corresponding to GEA grading for acne on the face [34] and score 2 corresponding to the evaluation of the mandibular region. Scores are not added together. For each patient, AFAST score 1 (GEA) and AFAST score 2 should be assessed by the same evaluator at each visit.

The other score we will use is the ECLA. The ECLA chart for evaluation of acne has been developed by six dermatologists (in private and hospital practice) [35]. It is composed of 3 factors: factor 1 (F1) counts the acne lesions on the face, factor 2 (F2) counts the acne lesions on the trunk and factor 3 (F3) counts the scars. In this study, only factor F2 will be used. Factor F2 assesses the extensive character of acne lesions in five defined areas: cervical area (F2N), chest area (F2C), back area (F2B) and arm area (F2A) according to a qualitative scale where 0 = absent, 1 = poor, 2 = medium and 3 = significant. It is completed by counting the nodules present in each area.

With regard to the study questionnaires, we will use the EQ-5D and the CADI. The EQ-5D questionnaire, validated in France [46], will be given to the patient at each visit to measure their quality of life. The EQ-5D consists of a questionnaire and a visual analogue scale. The questionnaire focuses on 5 dimensions: mobility, personal autonomy, current activities, pain/discomfort and anxiety/depression. For each of these dimensions, three answers are possible (EQ-5D-3 L), thus allowing for 243 health states. The CADI is a disease-specific questionnaire measuring disability induced by acne [47]. It is a well-known acne disability measure and has been used in some studies to assess the burden of living with acne on a patient’s experience of disability [48, 49]. The questionnaire was developed as a multidimensional measure related to the impact of acne [50]. It is a 5-item scale: questions 1 and 2 address the psychological and social consequences of acne in general; question 3 is targeted at those with acne of the chest or back; question 4 enquires about the patient’s psychological state; and question 5 asks for the patient’s (subjective) assessment of their current acne severity. The response to each question is scored from 0 to 3. CADI is validated by our team in French [51].

Bacterial and parasitic samples will be collected from the nodulocystic and pustular skin of the participants at D0 and M4, using an Eswab to be delivered in an AMIES transport environment (1 ml). The analysis will be centralized at Nantes University hospital and performed by Dr Stéphane Corvec.

### Plan to promote participant retention and complete {18b}

The patient will be given a diary at the randomization visit, to note their health resource consumption, medical compliance, concomitant treatments and AEs. The visits will be arranged every 2 months up to M6, where the main objective is set. Empty blisters will be collected and counted at each visit. At each visit, the drugs prescribed will be for 2 months (blinded allocation of spironolactone/doxycycline and a placebo plus concomitant topical therapy). At visit M2 (2 months after randomization), the physician or a member of their team will have to be vigilant and tell the patient to carefully follow the chronological order of treatments. Two envelopes will be given to the patient, one for M3 (blinded allocation of spironolactone or doxycycline) and one for M4 (blinded allocation spironolactone or a placebo in place of doxycycline). As aforementioned, the guidelines stipulate that treatment with doxycycline should not exceed 3 months. For this reason, patients in the doxycycline arm will be given a placebo during month 4 and the following months [41–43].

### Data management {19}

An eCRF will be drawn up for each participant. The participant will be identified using the first letter of the family name, the first letter of the first name, the centre number and the inclusion number, supplemented by the month and year of birth. This code should be the only information featured in the eCRF, enabling a retrospective link with the patient.

The investigator will also encode the patient data on any documents that may be in their possession (imaging, biology test reports, etc.) and attached to the eCRF. At the end of the study, the CRF database and safety database will be reconciled. This reconciliation will be performed before database locking. Similarly, an annual reconciliation will be carried out when updating the Annual Safety Report (ASR).

### Confidentiality {27}

Each patient’s medical data will only be provided to the Sponsor or any person duly authorized by the Sponsor and, where applicable, to authorized health authorities, under conditions of confidentiality. The Sponsor and the supervisory authorities may request direct access to medical records for the purposes of verification of the procedures and/or data in respect of the clinical trial, within the limits authorized by legislation and regulations. The data compiled during the trial may be processed electronically in compliance with the French Data Protection Agency (CNIL) requirements. CNIL is an independent French administrative regulatory body whose mission is to ensure that data privacy legislation is applied to the collection, storage and use of personal data.

### Plans for the collection, laboratory evaluation and storage of biological specimens for genetic or molecular analysis in this trial/future use {33}

At the end of the study, participants’ biological samples (bacterial strains) will be kept in anticipation of further scientific benefit, but naturally the participant’s written consent will be obtained. The samples will be stored in the biocollection under the responsibility of Prof. Dréno. This biocollection and its consent procedure have been registered under number DC-2011-1399 by the French Ethics Committee: CPP Ouest IV.

### Statistical methods

#### Statistical methods for primary and secondary outcomes {20a}

The primary efficacy variable is the percentage of success defined in “Background and rationale” (paragraph 2.1.2). The comparison between the two arms will be done using a Cochran–Mantel–Haenszel test checking for class of contraception (four strata). Modified intention to treat (ITTm) andlysis will be applied. That implies that to maintain the initial statistical power of the ITTm analysis, all randomized patients will be included in the final analysis, minus those who did not fulfil the inclusion criteria after randomization and those who never began their treatment.

All secondary objectives are expressed as a percentage or a status at a fixed time of follow up. General linear mixed models will be planned to take into account the repeated measurements collected on the same participant during their follow up. The Cox model with time-dependent covariates can also be used for secondary objectives for which a status is considered. Significance will be set at 5% in a bilateral situation.

For the cost-effectiveness analysis, 95% confidence intervals for differential (between arms) costs and QALYs and for the incremental cost-effectiveness ratio (ICER) will be estimated using the non-parametric bootstrap estimation procedure.

### Interim analyses {21b}

No interim analysis will be performed and no early stopping rule for futility is proposed.

### Methods for additional analyses (e.g. subgroup analyses) {20b}

An economic evaluation taking the form of a cost-effectiveness analysis (CEA) will be conducted to compare the efficiency of spironolactone and cycline treatment. The analysis will be conducted from the societal perspective, which means that costs to hospitals, the National Health Insurance System and patients will be considered, over a 6-month time horizon, to be consistent with the primary endpoint. The main judgement criterion will be the incremental cost-effectiveness ratio defined as a cost per QALY gained. To estimate QALYs, patients will be asked to fill in the EuroQol EQ-5D health-related quality of life questionnaire.

For the estimation of costs, resources consumed will be recorded prospectively in the patient’s diary for a period of 6 months following the start of the treatment. The main resources to be collected are in the patient diary are: * Medication * Ambulatory physician visits * Dermocosmetic care.

Unit costs will be estimated using conventional tariffs from the French National Health Insurance System to value ambulatory care consumption. Costs in each arm will be estimated by multiplying the amount of each type of resource consumed by their corresponding unit monetary value. In CEA, the outcomes of an intervention are evaluated in terms of the QALY, which is a numerical composite measure that encompasses information about the length of life and the health-related quality of life. They are computed by weighting each year by a corresponding quality of life factor, usually ranging from 0 (death) to 1 (perfect health). Quality of life weights will be obtained by asking patients to answer the EuroQol EQ-5D quality of life questionnaire at baseline and at 2, 4, and 6 months from baseline. The difference in QALYs between the two arms of the study will be calculated using analysis of area under the curve, with linear interpolation of utility scores between measurement time points.

The EQ-5D will also be applied at 9 and 12 months to evaluate quality of life up to M12, as a secondary objective, independently from the cost-effective analysis. The ICER (see below) comparing the spironolactone and cycline arms will be analysed using a non-parametric bootstrap approach to produce estimated confidence intervals at 95% over the mean ICER:

$$ \mathrm{ICER}=\left[{\mathrm{Costs}}_{\mathrm{Spironolactone}}-{\mathrm{Costs}}_{\mathrm{Cycline}}\right]/\left[{\mathrm{QALYs}}_{\mathrm{Spironolactone}}-{\mathrm{QALYs}}_{\mathrm{Cycline}}\right] $$

The cost-effectiveness of spironolactone will be assessed according to a willingness to pay of 50,000 euros. The probability of cost-effectiveness will also be assessed with several willingness to pay thresholds and will be represented in an acceptability curve. A sensitivity analysis will be performed in order to assess the robustness of the ICER result.

### Methods in analysis to handle protocol non-adherence and any statistical methods to handle missing data {20c}

All participants will receive the assigned study treatment until unacceptable toxicity, death, loss to follow up, withdrawal of consent or termination of the study by the Sponsor, in accordance with local standards of care. Unacceptable toxicity and death will be considered as failure for the primary criteria analysis. Loss to follow up, missing data for the primary criteria and early termination of the study by the Sponsor will be also considered as failures by the ITTm and not replaced.

Patients who did not fulfil the inclusion criteria after randomization and those who never began their treatment will be replaced. For the clinical secondary criteria analysis, patients with missing data will be withdrawn. Missing data in cost-effectiveness analysis will be handled according to a multiple imputation method in the ITTm sample. If a patient is released from the study (e.g. in the event of adverse reactions (ARs) preventing the continuation of the study), her data will not be collected, except for safety data (follow up of ARs or onset of ARs associated with the experimental treatment, based on pharmacokinetic data).

### Plans to give access to the full protocol, participant-level data and statistical code {31c}

According to French law, the results of the study will be published on the website of the regulatory authority. Data sharing will be between the investigators only. However, the datasets analysed during the current study will be available from the corresponding author on reasonable request.

## Oversight and monitoring

### Composition of the coordinating centre and trial steering committee {5d}

It has been possible to develop the protocol and carry out the trial thanks to an Executive Committee, which includes a Scientific Committee and a Steering Committee. The Scientific Committee was created and coordinated by Prof. B. Dreno. Its membership comprises a biostatistician and methodologist, the study coordinator, the health economist and the project manager of the clinical investigation centre (CIC1413). The Steering Committee is composed of the members of the Scientific Committee with the addition of the data management team, the study nurse who coordinates assistance for patient inclusion in the other centres and the monitoring Clinical Research Assistant (CRA). The Sponsor project manager coordinates this committee and drafts the “FASCE newsletter”, which provides, among other things, the latest news on patient inclusion and amendments to the protocol.

### Composition of the data monitoring committee, its role and reporting structure {21a}

The Data and Safety Monitoring Committee (DSMC) is an advisory committee responsible for reviewing the safety of a clinical trial for the Sponsor and the coordinator of the study. Its members, well-versed in the field of clinical trials (pathology and methodology), are not involved in the study. They are appointed for the duration of the study and undertake to take part in and observe data confidentiality. The members of the DSMC are selected collectively by the coordinator, Prof. B. Dreno and the Sponsor. The DSMC is convened at least once a year for safety analysis: the members receive the annual safety report and may be additionally consulted if a suspected unexpected serious adverse reaction (SUSAR) or serious adverse reaction (SAR) involves a specific analytical problem or in the event of any unresolved risk/benefit question arising during the course of the study.

### Adverse event reporting and harms {22}

Side effects reported in clinical trials and case series with spironolactone and the management of acne in women [22] are as follows: (1) the most common side effect in both the clinical trials and case series was menstrual irregularities: 38/264 (14.4%) in the clinical trials and 216/543 (39.8%) in the case series. Thee was no side effect apart from menstrual disturbances with an incidence above 5% in the randomized clinical trials or case series; (2) uncommon side effects (0.1–1.0%) were postural hypotension, depression, diarrhea, muscle pain, increased appetite, drowsiness, rashes/drug eruptions, chloasma-like skin pigmentation, polydipsia, weakness, edema of the legs, change in libido and palpitations. No women were reported to have elevated levels of potassium, and a recent multicentre study of 974 women [52] concluded that routine monitoring of serum potassium in healthy women taking spironolactone for acne is not necessary. Some investigators mentioned that certain side effects were considered beneficial: breast enlargement, reduced symptoms of premenstrual syndrome and less greasy skin and hair. The most frequent adverse reactions to cyclines are photosensitization, gastrointestinal disorders including gastric or esophageal pain, and nausea/vomiting*.*

Any adverse event, whether expected or unexpected, serious or not, must be collected real-time in the study eCRF. All SAEs, whether expected or unexpected, must be reported immediately (on the day of the investigator becoming aware of the event) to the Sponsor through the eCRF. The information mentioned on this form and on attached documents must be complete, accurate, clear (no abbreviations) and coded. Pregnancy, overdose, misuse, medication errors or potential medication errors and quality defects should be notified by the investigator to the Sponsor even if there is no associated adverse reaction.

### Frequency of and plans for trial conduct auditing {23}

An inspection or audit may take place as part of this study, performed by the Sponsor and/or by the regulatory authorities. Inspectors will check the documents, logistics, records and any other resources that the authorities consider to be associated with the clinical trial and that may be located at the trial site itself.

### Plans for seeking research ethics committee/institutional review board (REC/IRB) approval {24}

This clinical study was submitted to and approved by the southwest Ethical Review Board (*Comité de Protection des Personnes Sud-Ouest et Outre-mer III*) on 3 October 2017.

### Plans for communicating important protocol amendments to relevant parties (e.g. trial participants, ethical committees) {25}

The amended protocol should be a dated, updated version. If necessary, the information form and consent form should be amended. The updated protocol is at version 8 on 26 November 2019. All the submissions/declarations were made by the Sponsor Department at CHU Nantes to the French Regulatory Authority (ANSM) and the southwest Ethical Review Board (*Comité de Protection des Personnes Sud-Ouest et Outre-mer III*).

### Statement of who will have access to the final trial dataset, and disclosure of contractual agreements that limit such access for investigators {29}

The investigators will share the entirety of the final trial dataset.

### Disseminations plans {31a}

The trial results will be published in international dermatological, medical and scientific journals and presented at national and international conferences.

### Authorship eligibility guidelines and any intended use of professional writers {31b}

The investigators will follow the rules and guidelines of the International Committee for Medical Journal Editors (ICMJE) [53]. In practice, the Scientific Committee will be among the authors of the publication, as will the investigators who have included the most patients in the trial. The trial Sponsor and the French Ministry of Health, which provided the grant, must be cited in the publication.

## Discussion

Although it has been prescribed for more than 30 years in the USA as an acne treatment in women, spironolactone has no official labelling for acne anywhere in the world, which complicates its use by dermatologists treating this condition. Thus, if we demonstrate the superiority of spironolactone over cycline in the treatment of adult female acne, our randomized trial could have an impact on daily practice, leading the dermatologist to prescribe this treatment with the guarantee of an acceptable benefit/risk balance.

Moreover, beyond the demonstration of the superiority of spironolactone over cycline in the treatment of adult female acne, several collective benefits of this FASCE trial can be envisaged. First, this treatment will greatly improve the quality of life of these patients. This will have social and economic impacts given the substantial social and economic costs of acne vulgaris care. For example, in the USA, acne vulgaris has been responsible for more than 5 million physician visits [54].

Second, as aforementioned, the WHO is worried about the excessive use of antibiotics [31]. This study, if the results are conclusive, will allow physicians to choose spironolactone instead of antibiotics. This saving in antibiotic prescriptions will contribute to reducing the occurrence of antibiotic resistance.

Third, spironolactone can be used as an alternative to isotretinoin in women and this is of particular interest for two reasons: first, isotretinoin is associated with a strict programme of contraception that is often difficult for patients to follow; second, the peripheral hyperandrogenia that frequently occurs in women does not respond well to isotretinoin, with frequent relapses.

In view of all the potential benefits and impacts of this study, its results are eagerly awaited by dermatologists, and in particular by private dermatologists, who see these women suffering from acne in their clinical practice.

## Trial status

This trial is still ongoing; patient inclusion is not yet complete.

The updated protocol is version 8 on 26 November 2019.

The first patient was included on 31 January 2018.

Recruitment by the investigating centres is planned to continue until 30 June 2021.

## Supplementary information

**Additional file 1.** Informed Consent Form. The informed consent form given to each patient (French version).

## Data Availability

Data sharing is not applicable to this paper as no datasets were generated or analysed during the current study. The data from the completed trial will not be shared and will only be transmitted to the Sponsor. Data collected during the test may be processed electronically, in accordance with the requirements of CNIL (compliance with reference methodology MR001).

## Abbreviations

ACE: Angiotensin-converting enzyme
AFAST: Adult Female Acne Scoring Tool
ANSM: Agence Nationale de Sécurité du Médicament et des produits de santé
AR: Adverse reaction
ASR: Annual Safety Report
CADI: Cardiff Acne Disability Index
CEA: Cost-effectiveness analysis
CNIL: Commission Nationale de l’Informatique et des Libertés
CRA: Clinical Research Associate (monitor)
CRF: Case report form
DHEAS: Dehydroepiandrosterone
DHT: Dihydrotestosterone
DSMC: Data and Safety Monitoring Committee
ECLA: Echelle de cotation des Lésions d’Acné
eCRF: Electronic case report form
EQ-5D: Euroqol-5 dimensions questionnaire
GAGS: Global Acne Grading System
GCP: Good Clinical Practice
GEA: Global Evaluation Assessment
ICER: Incremental cost-effectiveness ratio
IGF 1: Insulin-like growth factor 1
IUD: Intra-uterine device
LH: Luteinizing hormone
NSAID: Non-steroidal anti-inflammatory drug
OC: Oral contraceptive
QALY: Quality-adjusted life year
QoL: Quality of life
RCT: Randomized controlled trial
SAE: Serious adverse event
WHO: World Health Organisation
