# Supplementary material for: FASCE, the benefit of spironolactone for treating acne in women: study protocol for a randomized double-blind trial
Source: Trials. 2020 Jun 25;21:571. doi: 10.1186/s13063-020-04432-w (PMC7318446; doi:10.1186/s13063-020-04432-w)
Supplement: Supplementary file 1 — Additional file 1. Informed Consent Form. The informed consent form given to each patient (French version). [file 13063_2020_4432_MOESM1_ESM.docx]

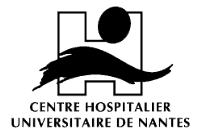


**Note d’information pour la participation à la recherche**

  Etude randomisée en double aveugle de l’intérêt de la spironolactone pour le traitement de l’acné de la femme adulte

**Titre abrégé : FASCE**

***Promoteur : CHU de Nantes***

***N° EudracT/ Enregistrement :* 2017-001392-22**

Madame,

Dans le cadre de la prise en charge de votre maladie, l’acné, nous vous proposons de participer à une recherche interventionnelle nommée « FASCE». Cette recherche a pour but de tester la spironolactone dans le traitement de l’acné de la femme adulte.

Le CHU de Nantes est le promoteur de cette recherche, c’est-à-dire qu’il en est responsable et qu’il l’organise.

Votre participation à la recherche, au cas où vous donneriez votre accord, ne pourra vous être confirmée qu’à la condition que vous remplissiez tous les critères d’inclusion pour participer à cette recherche.

# COMMENT VOUS DECIDER ?

- Votre dermatologue*,* c’est-à-dire l’investigateur de l’étude, vous a donné des explications. Elles sont résumées dans ce document intitulé « note d’information ». Nous vous invitons à le lire attentivement avant de vous décider. Vous avez un délai de réflexion de 7 jours avant de vous décider.
- Si vous décidez de participer à cette recherche, nous vous demanderons de dater et signer une attestation de consentement. Cette attestation sera aussi signée par l’investigateur. Cette signature confirmera que vous êtes d’accord pour participer à la recherche. Votre signature est indispensable, ainsi que celle de l’investigateur. Même après avoir signé pour donner votre accord de participation, vous garderez le droit d’interrompre à tout moment votre participation sans avoir à vous justifier.

# QUE SAIT-ON DEJA SUR LE PRODUIT A L’ETUDE ?

La spironolactone appartient à la classe des médicaments appelés diurétiques (des traitements qui augmentent l'excrétion de l'urine). Elle possède l’autorisation de mise sur la marché (AMM) en France depuis 1982 et aux Etats-Unis depuis 1985, pour soigner l'œdème (une rétention d'eau dans les tissus de l'organisme) qui peut être provoqué par une insuffisance cardiaque congestive, une cirrhose du foie ou un syndrome néphrotique. Elle s'utilise aussi dans le traitement d'une pression artérielle élevée et d'un hyperaldostéronisme primitif (une affection résultant d'une surproduction d'aldostérone par les glandes surrénales) dont elle sert d'ailleurs à poser le diagnostic.

La spironolactone est également indiquée pour soigner ou prévenir une hypokaliémie (trop peu de potassium dans le corps) quand d'autres méthodes thérapeutiques ne conviennent pas. La spironolactone agit en aidant le corps à éliminer l'excès d'eau et de sel, mais elle réduit la perte de potassium du corps.

La spironolactone, de par sa composition, est aussi un anti-androgène. Elle a de ce fait été utilisée pour le traitement de l’hirsutisme. L’hirsutisme se traduit chez la femme, par une pilosité excessive de type masculine dûe à une augmentation de la sécrétion d’androgènes. L’hirsutisme s’accompagne souvent d’une acné. Or quand la spironolactone a été utilisée pour le traitement de cette pathologie, la pilosité et l’acné ont diminué.

Actuellement des études sur quelques patientes en Europe et aux Etats-Unis, ont montré de bons résultats quant au traitement de l’acné adulte. Dans le département de dermatologie du Pr Dréno, au CHU de Nantes, la spironolactone a été utilisée pour traiter efficacement environ 200 patientes. Mais aucune étude scientifique n’a été menée pour comparer la spironolactone au traitement traditionnel de l’acné de la femme adulte : la doxycycline.

La doxycycline fait partie des tétracyclines, antibiotiques utilisés pour le traitement de l’acné.

# QUEL EST LE BUT DE CETTE RECHERCHE ? QUEL EST SON DEROULEMENT GENERAL ?

La recherche est réalisée sous la responsabilité du Centre Hospitalier Universitaire de Nantes. Cette étude nationale aura lieu dans plusieurs centres de référence de l’acné.

Les hormones semblent jouer un rôle de premier plan dans l’acné chez la femme adulte, ce qui explique les poussées lors des variations hormonales. Un traitement hormonal anti-androgène peut être proposé dans l’acné car la glande sébacée à l’origine de la peau grasse de l’acné a des récepteurs aux androgènes dont l’activation favorise l’acné. Or la spironolactone est un anti-androgène. Le but de cette étude est de démontrer que la spironolactone peut être un traitement de l’acné de la femme adulte et donc de la comparer au traitement conventionnel de l’acné, la doxycycline.

- L’étude sera menée en double-aveugle durant les six premiers mois de la prise en charge, cela signifie que ni vous ni l’investigateur ne saurez si vous recevez le produit à l’étude, la spironolactone, ou bien le comparateur, la doxycycline. L’étude sera randomisée, c’est-à-dire qu’un tirage au sort réalisé informatiquement déterminera quel traitement vous recevrez. Ceci permettra à l’investigateur d’être plus objectif concernant les effets du produit à l’étude.
- L’étude se déroulera sur 12 mois. La spironolactone peut être prescrite pendant 12 mois, cependant la doxycycline ne peut être donnée en traitement de l’acné que pendant 3 mois. Les patientes qui auront la doxycyline comme traitement auront 3 mois de traitement puis 3 mois de placebo pour maintenir l’aveugle de cette étude. Un placebo est une substance qui n’a pas d’effet, par exemple un granule de sucre.

# QU’ARRIVERA-T-IL PENDANT LA RECHERCHE ? QU’AUREZ-VOUS A FAIRE ?

**Votre participation éventuelle à cette recherche durera 12 mois**

Votre accord pour participer à cette recherche demandera de votre part que vous vous engagiez pendant toute la durée de la recherche à :

- Vous rendre à toutes les visites prévues dans le protocole
- La visite de sélection : vérification des critères d’inclusion et de non-inclusion, information de la patiente, collecte d’informations médicales, remise de la carte de participation au protocole. Une prise de sang sera réalisée afin d’effectuer : un test de grossesse (les traitements de l’étude sont contre-indiqués en cas de grossesse), un ionogramme (examen très courant qui analyse la concentration en électrolytes, ici le potassium et le sodium) et un dosage AMH (hormone anti-mullerienne afin de déterminer un éventuel syndrome d’ovaires polykistiques, non compatible avec les traitements à l’étude).

Le consentement pourra être signé le jour de la visite de sélection ou ultérieurement mais impérativement avant la visite de randomisation.

La visite de randomisation : tirage au sort informatique pour déterminer quel traitement vous aurez : soit la doxycycline (100mg/jour) soit la spironolactone (150mg/jour). Dans tous les cas, un traitement local (une pommade) de peroxyde de benzoyle, classiquement donné pour l’acné, vous sera donné en complément, pour traiter les lésions. Un examen clinique sera fait au cours duquel le médecin notera les lésions d’acné sur votre visage et sur la partie haute de votre corps. Deux prélèvements acnéiques seront réalisés afin de déterminer la nature bactériologique et parasitologique de vos lésions :

- - - Le prélèvement bactérien consiste à réaliser des rotations sur un bouton d’acné pendant une durée de 50 secondes avec un écouvillon (une sorte de coton tige).
    - Le prélèvement parasitologique consiste à prélever un échantillon d’un bouton d’acné en appliquant un morceau de Scotch sur le bouton puis en le décollant.

Vous devrez remplir 2 questionnaires de qualité de vie (environ 5 minutes chacun).

Un carnet patient vous sera remis pour suivre la bonne prise des médicaments donnés, noter vos éventuels problèmes de santé et vos traitements associés. Afin de mesurer l’impact sur le plan économique des deux traitements à l’étude, il vous sera également demandé de recueillir, sur les 6 premiers mois de traitement uniquement, le nombre de consultations médicales ainsi que les produits cosmétiques utilisés et leur fréquence.

Après le début du traitement, vous aurez une visite tous les 2 mois pendant 6 mois et une tous les 3 mois pendant les 6 mois suivants.

- Visites de suivi à M2, M4, M6 et M9 :

Un examen clinique sera fait au cours duquel le médecin notera les lésions d’acné sur votre visage et sur le haut de votre corps. Vous devrez remplir 2 questionnaires de qualité de vie (environ 5 minutes chacun).

Deux prélèvements acnéiques identiques à ceux de la visite de randomisation seront réalisés à M4.

Une prise de sang de 500 µl aura lieu à M2, M4 et M9 pour la réalisation d’un ionogramme. Les valeurs biologiques du ionogramme (potassium, sodium) seront comparées aux valeurs de la visite de sélection afin de mesurer l’impact éventuel des traitements à l’étude sur ces valeurs.

Vous devrez rapporter votre carnet patient à chaque visite afin que les données soient reportées.

Lors de la visite M6, une fois que le médecin investigateur aura évalué l’efficacité du traitement, il prendra connaissance du traitement qui vous a été remis et vous en informera.

Si le traitement qui vous a été remis est la spironolactone et que votre acné a répondu à ce traitement, vous poursuivrez celui-ci au moins jusqu’à la visite suivante.

Si le traitement qui vous a été remis est la doxycycline et que votre acné a répondu à ce traitement, vous ne poursuivrez que le traitement local (pommade) au moins jusqu’à la visite suivante. En effet, le traitement antibiotique ne doit pas dépasser 3 mois.

En cas d’échec de traitement, votre participation à l’étude s’arrêtera et votre médecin investigateur vous prescrira un traitement alternatif selon sa pratique habituelle.

Il en sera de même lors de la visite M9.

- La visite de fin de la recherche M12

Un examen clinique sera fait au cours duquel le médecin comptera les lésions d’acné sur votre visage et sur le haut de votre corps.

Vous devrez remplir 2 questionnaires de qualité de vie (environ 5 minutes chacun).

Le carnet patient devra être remis à votre médecin-investigateur.

Un prélèvement sanguin pour la réalisation d’un test de grossesse sera à nouveau effectué en fin d’étude ou dès que votre participation s’arrêtera (les traitements de l’étude sont contre-indiqués en cas de grossesse).

Tout au long de l’étude, votre médecin-investigateur vous demandera de bien suivre les recommandations habituelles dans le traitement de l’acné:

- L’utilisation d’un gel nettoyant ou d’un pain dermatologique avec un pH autour de 5 qui est le pH de la peau et une crème hydratante non-comédogène le matin sur le visage sans frotter,
- L’utilisation d’une protection solaire, de produits dermo-cosmétiques (maquillage…) non-comédogènes, adaptés aux peaux acnéiques.

L’investigateur ou un membre de son équipe vous fournira toutes les explications nécessaires et répondra à toutes vos questions.

# QUELS SONT LES RISQUES ?

Cette recherche ne présente que des risques et contraintes minimes par rapport à la prise en charge normale qui vous serait proposée si vous ne participiez pas à la recherche.

- Principaux effets indésirables des traitements utilisés dans cette étude :

- spironolactone : troubles menstruels, intolérance digestive, crampes dans les jambes, problèmes de sommeil, éruption cutanée, tension des seins.

Pour les femmes dont l’acné est traitée par la spironolactone ce sont principalement les troubles menstruels qui sont rapportés.

- doxycycline: photosensibilité, éruption cutanée, mal de tête, nausée, réaction anaphylactique (urticaire, œdème, difficultés respiratoires…), troubles digestifs, douleurs gastriques ou oesophagiennes

- traitement local (peroxyde de benzoyle) : sensation de brûlure, peau asséchée, exfoliation de la peau

- Risques liés aux prélèvements : les prélèvements sanguins peuvent éventuellement former un hématome.
- Risques liés aux situations particulières : en cas de grossesse, la spironolactone peut induire la féminisation du fœtus masculin. Par ailleurs il est préférable de ne pas utiliser la doxycycline pendant le premier trimestre de la grossesse car il y a un risque d’exposer le fœtus à la coloration des dents de laits. L'utilisation de ce médicament est contre-indiquée pendant les deuxième et troisième trimestres de grossesse. Une méthode de contraception efficace (pillule, stérilet…) devra être utilisée tout au long de l’étude.

# En cas de grossesse en cours d’étude, vous devrez informer immédiatement votre médecin investigateur.

# QUELS SONT LES BENEFICES QUE VOUS POUVEZ ESPERER ?

- Bénéfice personnel thérapeutique attendu : si vous êtes randomisée dans le bras « spironolactone » le bénéfice attendu est une efficacité supérieure aux antiobiotiques sur les lésions d’acné (la doxycyline). Si vous êtes randomisée dans le bras doxycycline le bénéfice attendu est d’être traité par le traitement de référence qui a déjà montré son efficacité.
- Bénéfice collectif attendu : la participation volontaire à une recherche interventionnelle est utile à tous.

Si la supériorité de la spironolactone sur la doxycycline (traitement courant) est démontrée dans le traitement de l’acné, sa prescription sera généralisée et prise en charge par la sécurité sociale.

De plus, l’Organisation Mondiale de la Santé s’inquiète de l’utilisation excessive d’antibiotiques qui conduiront en 2050 à des bactéries résistantes. Pouvoir soigner l’acné, sans utiliser d’antibiotiques devient un objectif de Santé Publique mondiale.

# QUELLES SONT LES ALTERNATIVES ?

Si vous ne souhaitez pas participer, le médecin-investigateur décidera avec vous de la meilleure solution pour traiter votre acné.

# QUE SE PASSERA-T-IL A LA FIN DE LA RECHERCHE, SI LA RECHERCHE S'ARRETE OU SI VOUS DECIDEZ D’INTERROMPRE VOTRE PARTICIPATION ?

La recherche peut être interrompue à tout moment:

- par les autorités de santé,
- du fait du promoteur, le CHU de Nantes : si un élément nouveau survient, l’investigateur en sera informé et il vous transmettra alors les éléments susceptibles de modifier votre participation.
- du fait de l’investigateur, pour des raisons médicales vous concernant : il peut décider à tout moment d’arrêter l’administration du produit à l’étude (par exemple à cause d’un effet secondaire ou d’une évolution de votre état de santé) et vous en informera.
- par vous-même : si vous décidez de participer à cette recherche, il s’agira d’un acte volontaire. Vous pourrez à tout moment décider d’arrêter votre participation, sans pénalité ni préjudice. Dans ce cas, vous devez informer l’investigateur de votre décision.

Quelle que soit la raison de votre interruption, l’investigateur vous informera alors des mesures à suivre. Si vous étiez sous spironolactone, ce médicament n’ayant pas l’autorisation de mise sur le marché pour l’acné, le traitement avec cette molécule ne pourra pas être poursuivi.

Mais dans tous les cas, la qualité de votre prise en charge ne sera pas diminuée.

# AUREZ-VOUS DES FRAIS SUPPLEMENTAIRES ?

Votre participation à cette recherche n'engendrera pour vous aucun frais supplémentaire par rapport à ceux que vous auriez dans la prise en charge habituelle de cette maladie.

La visite à M2 est réalisée en plus du suivi habituel, vos transports seront donc remboursés sur une base forfaitaire de 67.5€ pour cette visite.

# QUELS SONT VOS DROITS PENDANT LA RECHERCHE ?

- **Secret professionnel**

Le personnel impliqué dans la recherche est soumis au secret professionnel, tout comme votre médecin traitant.

Sauf avis contraire de votre part, votre médecin traitant pourra être informé de votre participation.

- **Accès aux données vous concernant - Traitement des données**

Dans le cadre de cette recherche, un traitement informatique de vos données personnelles va être mis en œuvre : cela permettra d’analyser les résultats de la recherche et de remplir l’objectif de la recherche.

Pour cela, les données médicales vous concernant (et les données relatives à vos habitudes de vie), seront transmises au Promoteur de la recherche (CHU de Nantes) ou aux personnes ou sociétés agissant pour son compte. Ces données seront identifiées par un numéro de code et vos initiales. Ces données seront susceptibles d’être exploitées dans le cadre de publications ou de communications ; dans ce cas, votre anonymat sera préservé.

Si vous décidez de retirer votre consentement pour participer à la recherche, les données obtenues avant que celui-ci n'ait été retiré pourront être utilisées, sauf opposition expresse de votre part. Les données recueillies après le retrait de votre consentement ne seront pas utilisées pour cette recherche et resteront destinées à l’usage strict du soin.

Ces données pourront également, dans des conditions assurant leur confidentialité, être transmises aux autorités sanitaires habilitées. Conformément aux dispositions de la loi relative à l’informatique aux fichiers et aux libertés (loi modifiée du 6 janvier 1978), vous disposez d’un droit d’accès et de rectification. Vous disposez également d’un droit d’opposition à la transmission des données couvertes par le secret professionnel susceptibles d’être utilisées dans le cadre de cette recherche et d’être traitées.

Ces données pourront être utilisées lors de recherches ultérieures exclusivement à des fins scientifiques. Vous pouvez retirer votre consentement à cette utilisation ultérieure ou exercer votre faculté d'opposition à tout moment.

Ces droits s’exercent auprès de l’investigateur qui vous suit dans le cadre de la recherche et qui connaît votre identité.

Vous pouvez également accéder directement ou par l’intermédiaire d’un médecin de votre choix à l’ensemble de vos données médicales en application des dispositions de l’article L 1111-7 du Code de la Santé Publique.

- **Accès aux résultats globaux de la recherche**

A la fin de la recherche, et à votre demande, vous pourrez être informé(e) par l’investigateur des résultats globaux de cette recherche (dès qu’ils seront disponibles).

# QUELLES SONT VOS OBLIGATIONS PENDANT LA RECHERCHE ?

- **Vos obligations**

Vous devez informer l’investigateur de tous les médicaments que vous prenez.

Vous devez aussi l’informer immédiatement de tout évènement ou effet indésirable éventuellement rencontré au cours de votre participation à la recherche.

Vous devez vous rendre aux visites prévues.

- **Protection sociale**

Pour pouvoir participer à cette recherche vous devez être affilié(e) ou bénéficier d’un régime de sécurité sociale *(CMU acceptée)*.

- **Modalités de participation à une autre recherche**

Pour l’élimination de la molécule de votre organisme un délai de deux semaines est nécessaire avant de participer à une autre recherche interventionnelle

# LE CADRE REGLEMENTAIRE

**Cette recherche est conforme :**

- Aux articles L. 1121-1 à L. 1126-12 du code de la santé publique relatifs aux recherches impliquant la personne humaine

- A la loi « Informatique et Libertés » du 6 janvier 1978 modifiée.

Vous pouvez retrouver tous ces textes sur le site <http://www.legifrance.gouv.fr>

**Conformément aux dispositions réglementaires :**

- Le CHU de Nantes organise cette recherche en tant que « promoteur ». Il a souscrit un contrat d’assurance garantissant sa responsabilité civile et celle de tout intervenant auprès de la compagnie HDI (Contrat n° 0100755914039 170020).
- Cette recherche a reçu l’avis favorable du Comité de Protection des Personnes Sud-Ouest et Outre-mer III le 03/10/2017. La recherche a aussi reçu l’autorisation de l’ANSM (Agence Nationale de Sécurité du Médicaments et des Produits de Santé), le 21/08/2017.

# QUEL EST LE DEVENIR DE VOS ECHANTILLONS PRELEVES AU COURS DE LA RECHERCHE ?

A la fin de la recherche et si vous en êtes d’accord, les échantillons biologiques résultant de votre prise en charge seront conservés dans une biocollection. Celle-ci sera gardée pour d’éventuels projets de recherche et ainsi vos échantillons pourront être utilisés. Un formulaire de consentement (différent de celui portant sur la recherche expliquée dans la présente note) va vous être soumis. Votre médecin investigateur ou un membre de son équipe vous fournira toutes les explications nécessaires et répondra à toutes vos questions. Si vous êtes d’accord pour que vos échantillons soient conservés dans cette biocollection, vous devrez signer le formulaire.

# EN RESUME…

Votre participation à cette recherche est libre. Vous pouvez refuser de participer à cette recherche.

De plus, vous pouvez à tout moment vous retirer de cette recherche, sans préjudice.

Si vous décidez de refuser de participer à la recherche ou si vous décidez d’arrêter votre participation pendant la recherche :

- cela n’aura aucune conséquence sur la qualité des soins qui vous seront donnés
- vous devez simplement en informer l’investigateur.

Lorsque vous aurez lu cette note d’information et obtenu les réponses aux questions que vous vous posez en interrogeant l’investigateur (Dr/Pr………………..………………..), il vous sera proposé, si vous en êtes d’accord, de donner votre consentement écrit en signant le formulaire préparé à cet effet.

Votre participation à la recherche, au cas où vous donneriez votre accord, ne pourra vous être confirmée qu’à la condition que vous remplissiez tous les critères d’inclusion pour participer à cette recherche (notamment, test de grossesse et dosage AMH négatifs).

Si le nombre de personnes prévues dans l’étude a été atteint, vous pourrez finalement ne pas être incluse dans l’étude même après avoir signé votre consentement. Dans ce cas précis, vous ne serez plus suivie dans le cadre de l’étude et les données vous concernant ainsi que vos échantillons sanguins le cas échéant seront détruits. Cela ne portera, en aucun cas, préjudice sur la qualité de votre prise en charge médicale.

Vous pouvez prendre votre temps avant de nous donner votre réponse.

Vous avez 7 jours pour prendre votre décision, à partir de la remise de cette note d’information.

Au cours de ce délai de réflexion, vous pouvez bien entendu continuer par téléphone à poser toutes les questions que vous souhaitez à l’investigateur (Dr/Pr.…………………….. tél………………………..).

Nous vous prions d’agréer, Madame, l’expression de nos sentiments les plus respectueux.

Pr Dréno, médecin et investigateur coordonnateur,

et toute l’équipe médicale en charge de cette recherche

*Vous devez conserver un exemplaire de ce document.*

**ANNEXES**

# ANNEXE 1 : LES CONTACTS IMPORTANTS :

**Le promoteur de la recherche :**

CHU de Nantes, direction de la recherche

🖂 5 allée de l’Ile Gloriette, 44093 NANTES Cedex 1

🕿 02 53 48 28 35

**L’investigateur coordonnateur de la recherche :**

Pr Brigitte Dréno

PU-PH, cancéro-dermatologie,

Hôtel Dieu, 1, place Alexis Ricordeau 44 000 Nantes

🕿 : 02 40 08 31 18

brigitte.dreno@wanadoo.fr
